# Editorial: Epigenetics in Cancer: Mechanisms and Drug Development

**DOI:** 10.3389/fgene.2022.831704

**Published:** 2022-06-28

**Authors:** Huiqing Yuan, Yongmei Huang, Susu Tao, Biaoru Li, Zhenhua Xu, Yi Qi, Binhua Wu, Hui Luo, Xiao Zhu

**Affiliations:** ^1^ School of Laboratory Medicine and Biomedical Engineering, Hangzhou Medical College, Hangzhou, China; ^2^ The Marine Biomedical Research Institute, Guangdong Medical University, Zhanjiang, China; ^3^ Southern Marine Science and Engineering Guangdong Laboratory (Zhanjiang), Zhanjiang, China; ^4^ The Key Lab of Zhanjiang for R&D Marine Microbial Resources in the Beibu Gulf Rim, Guangdong Medical University, Zhanjiang, China; ^5^ Cancer Center, Medical College of Georgia, Augusta University, Augusta, GA, United States; ^6^ Center for Cancer and Immunology, Children’s National Health System, Washington, DC, DC, United States

**Keywords:** cancer therapy, epigenetic drugs, DNA methylation, histone deacetylation, tumor inhibitors

This research topic “*Epigenetics in Cancer: Mechanisms and Drug Development*” consists of 14 articles contributed by more than 120 authors in the fields of cancer epigenetics and therapeutics. The topic enumerates collecting different research directions including transcription and chromatin roles in gene regulation, DNA modifications, RNA epigenetics, non-coding RNA, and epigenomic methods. In addition to bringing the newest findings on epigenetic mechanisms, a special focus will be given to novel and promising therapeutic drugs aimed at reversing specific epigenetic alterations.

In recent years, new targets of tumor immunotherapy have been found. For example, a checkpoint with forkhead associated and ring finger domain (*CHFR*) is one of the keys to immune checkpoints, and its activity is lost through promoter hypermethylation or mutation in many tumor and cancer cell lines. Chen et al. demonstrated that the epigenetic characteristics of the *CHFR* gene were a new prognostic feature. Dong et al. discussed the latest findings of the potential application of PIWIL1 in chemotherapy resistance of tumors through multiple signaling pathways.

The occurrence of cancer is related to the abnormal expression of many genes. Opioid binding protein/cell adhesion molecule-like (*OPCML*) is a protein-coding gene that has been associated with a variety of cancers, including ovarian cancer. Shao et al. observed that the DNA methylation level of the *OPCML* promoter region CG25853078 was positively correlated with its expression.

C-terminal Src kinase (Csk) and Csk homologous kinase (Chk) are the main endogenous inhibitors of Src family kinases (SFK) (Chueh et al., 2021). Zhu et al. found that increased DNA methylation levels may be caused by increased DNMT levels, leading to decreased expression of CHK mRNA and CHK protein and promoting the increase of carcinogenic characteristics of colon cancer cells. Epigenetic regulation of the CHK expression in colon cancer cells has significant biological effects, including cell proliferation, wound healing, and cell invasion.

In epigenetic modification, mRNA modification plays the same role as DNA methylation. Scientists have identified more than 100 chemical modification methods of RNA, among which N6-methyl adenine (m6A) accounts for 80% of RNA methylation modification (Zhou et al., 2020). M6A methylation modification has been proven to be reversible, which is controlled by methyltransferases (writers), methylated readers, and demethylases (Tan et al., 2021). The fat and obesity-related protein (FTO) has been identified as the first m6A methylase inhibitor and has been one of the most attractive target proteins for the development of m6A methylase inhibitors to treat cancer (Lu et al.).

More than ten kinds of posttranslational modifications (PTM) can occur on histones entangled with DNA, including methylation, acetylation, propionylation, phosphorylation, and ubiquitination. The most common ones are methylation and acetylation. Histone methylation is regulated by histone methyltransferases (HMTs) and histone demethylases (HDMs) (He and Lomberk). HMTs and HDMs balance each other to maintain histone methylation levels, and their imbalance may promote cancer. Acetylation is regulated by HATs and histone deacetylases (HDACs) (Zhang et al.; Li et al., 2022).

Abnormal DNA methylation has become a recurrent carcinogenic event (Liang et al., 2021). Zhu et al. emphasized that genetic aberrations rather than phenotypes (DNA methylation) can be targeted by identifying the molecular basis of carcinogenesis. In addition, although the restoration of the epigenome to normal is seen as a therapeutic strategy, it has not yet been proven to be the primary mechanism of treatment. It is necessary to create drugs that interfere with adaptive mechanisms, and this method is increasingly being proven.

In conclusion, the “*Epigenetics in Cancer: Mechanisms and Drug Development*” research topic highlights the importance of developing novel epigenetic targets for cancer therapy.
